# Supplementary material for: Splice-Junction-Based Mapping of Alternative Isoforms in the Human Proteome
Source: Cell Rep. Author manuscript; Available in PMC 2020 Jan 15. (PMC6961840; doi:10.1016/j.celrep.2019.11.026)
Supplement: 2 [file NIHMS1546469-supplement-2.zip › DF1/PXD006675/RightVentricle/RightVentricle_8_CLTCL1_LQEHFQLQNLGINPANIGFSTLTMESDK.pdf]

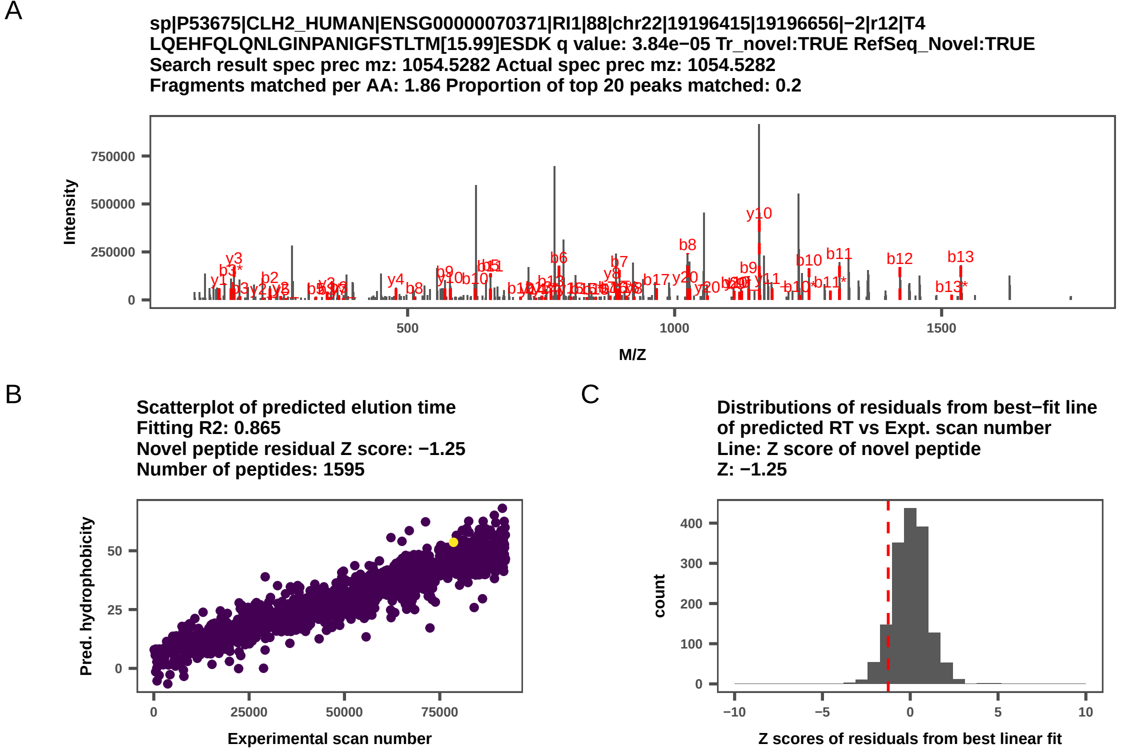

A
sp|P53675|CLH2_HUMAN|ENSG00000070371|RI1|88|chr22|19196415|19196656|−2|r12|T4
LQEHFQLQNLGINPANIGFSTLTM[15.99]ESDK q value: 3.84e−05 Tr_novel:TRUE RefSeq_Novel:TRUE
Search result spec prec mz: 1054.5282 Actual spec prec mz: 1054.5282
Fragments matched per AA: 1.86 Proportion of top 20 peaks matched: 0.2
Intensity
750000
500000
250000
0
500
1000
1500
M/Z
y10
b8
b10
b11
b12
b13
b13*
b6
b7
b9
y20
b17
y4
y3
b3*
b2
y1
B
Scatterplot of predicted elution time
Fitting R2: 0.865
Novel peptide residual Z score: −1.25
Number of peptides: 1595
Pred. hydrophobicity
50
25
0
0
25000
50000
75000
Experimental scan number
C
Distributions of residuals from best−fit line
of predicted RT vs Expt. scan number
Line: Z score of novel peptide
Z: −1.25
count
400
300
200
100
0
−10
−5
0
5
10
Z scores of residuals from best linear fit
